# Association between Cognitive Function and Depression with Human T-Cell Lymphotropic Virus 1 Seropositivity and Serointensity in UK Adults

**DOI:** 10.3390/pathogens10111409

**Published:** 2021-10-30

**Authors:** Lance D. Erickson, Dawson W. Hedges, Bruce L. Brown, Bradley Embley, Shawn D. Gale

**Affiliations:** 1 Department of Sociology, Brigham Young University, Provo, UT 84602, USA; lance_erickson@byu.edu; 2 Department of Psychology, Brigham Young University, Provo, UT 84602, USA; dawson_hedges@byu.edu (D.W.H.); bruce_brown@byu.edu (B.L.B.); 3 The Neuroscience Center, Brigham Young University, Provo, UT 84602, USA; bradleyembley@gmail.com

**Keywords:** human T-cell lymphotropic virus 1, HTLV-1, cognition, depression, UK Biobank

## Abstract

Several viral, bacterial, and parasitic diseases have been associated with cognitive function and neuropsychiatric outcomes in humans, including human T-cell lymphotropic virus 1 (HTLV-1). In this study, we sought to further generalize previously reported associations of cognitive function and depression with HTLV-1 seropositivity and serointensity using a community-based sample of adults aged approximately 40 to 70 years (mean = 55.3 years) from the United Kingdom. In this sample, the results of adjusted linear regression models showed no associations of HTLV-1 seropositivity or serointensity with reasoning, pairs-matching, or reaction-time cognitive tasks or with depression. In addition, neither age, sex, educational attainment, nor income moderated associations of HTLV-1 seropositivity or serointensity with cognitive function or depression. In this middle-aged to older middle-aged adult community sample, HTLV-1 seropositivity and serointensity do not appear to be associated with reasoning, pairs-matching, and reaction-time tasks or with depression.

## 1. Introduction

Several viral, bacterial, and parasitic diseases appear to be associated with cognitive function and dementia [1,2,3,4,5,6] and with neuropsychiatric disease in humans [7,8,9]. Among the infectious diseases associated with cognitive function and with neuropsychiatric outcomes in humans is human T-cell lymphotropic virus 1 (HTLV-1) [10,11].

HTLV-1 is a retrovirus that infects approximately 5 to 10 million people throughout much of the world, although this likely underestimates the actual number of people infected with HTLV-1 [12]. Transmission of HTLV-1 is typically through contaminated blood, by sexual transmission, or from mother to child via breastfeeding [12,13]. Because of its modes of transmission, the prevalence of HTLV-1 increases with age [12]. HTLV-1 is endemic in many regions, including Southwestern Japan, parts of the Middle East, the Caribbean, parts of South America including Brazil, sub-Saharan Africa, Australo-Melanesia, and Romania, although HTLV-1 infection can occur throughout much of the world [12,14]. In contrast, HTLV-1 seroprevalence is low in the United Kingdom [12].

In addition to its association with adult T-cell leukemia [13], HTLV-1 is associated with several neurologic diseases including polymyositis, polyneuropathy, dysautonomia [15], motor-neuron disease [16], and HTLV-1-associated myelopathy and tropical spastic paraparesis (HAM/TSP) [17], the last of which occurs in approximately 0.25 percent to 3.8 percent of all people with HTLV-1 [15] and which despite its name can occur in non-tropical regions [18]. HTLV-1 is also associated with some inflammatory diseases, such as arthritis and Sjögren’s syndrome [13] and can cause chronic encephalomyelopathy. Despite these associations with neurological diseases, most cases of HTLV-1 appear to be asymptomatic [15]. HTLV-1 also appears to result in elevated cytokines, chemokines, and other elements of the immune response [15].

In addition to its association with HAM/TSP and other diseases including encephalomyelopathy, and its possible relation to elevations in cytokines and chemokines [15], emerging findings show an association between HTLV-1 and cognitive function. In one study from Brazil that included 83 patients recruited from rehabilitation hospitals (mean age 55.3 years), all of whom had HAM/TSP, and 88 healthy controls, HTLV-1 infection was associated with decreased global cognition and with decreased executive function, an association the authors thought was possibly related to interleukin 6 [10]. Another study, also from Brazil, compared healthy controls to asymptomatic HTLV-1 patients, symptomatic HTLV-1 patients but without HAM/TSP, and HTLV-1 patients with HAM/TSP. This study found that while there were no differences in screening cognitive measures between groups, the combined overall HTLV-1 group had worse episodic memory and executive function, albeit with small effect sizes and worse fine-motor skills with large effect sizes compared to the control group. In addition, there were no differences in cognitive function between the HTLV-1 subgroups [17], findings suggesting that cognitive impairment in HTLV-1 can occur in the absence of HAM/TSP and in asymptomatic HTLV-1. In another study from Brazil [19], both asymptomatic HTLV-1 patients and HTLV-1 patients with HAM/TSP had worse cognitive function compared to healthy controls on some but not all of the assessed cognitive measures. However, only the group with HTLV-1 and HAM/TSP had major neurocognitive disorders, whereas the asymptomatic HTLV-1 group had only minor neurocognitive disorders [19], suggesting that asymptomatic HTLV-1 might possibly be associated with less cognitive dysfunction than HTLV-1 with HAM/TSP. In a study using a sample from an endemic region in Japan, HTLV-1 was associated with vascular dementia, but not with Alzheimer’s disease, providing further evidence that HTLV-1 is adversely associated with cognitive function. In this same study, HTLV-1 also was associated with ventricular enlargement and perivascular lucency [20].

In addition to its association with worse cognitive function, HTLV-1 also has been associated with depression and anxiety. A cross-sectional study from Brazil that investigated 20 patients with HAM/TSP, 38 asymptomatic HTLV-1 patients, and 340 healthy controls found that both HAM/TSP and asymptomatic HTLV-1 were associated with depression and anxiety [11], although the analyses in this study were not adjusted for covariates that could potentially influence the association between HTLV-1 and either depression or anxiety. Another study from Brazil compared levels of depression and anxiety between 63 patients with HAM/TSP and 67 patients with asymptomatic HTLV-1, finding that the patients with HAM/TSP had more depression and anxiety than the asymptomatic HTLV-1 group. This study did not include a non-HTLV-1 control group [21]. In a study from the United States, however, involving 155 HTLV-1 seropositive patients and 799 seronegative controls, there were no statistically significant associations between HTLV-1 and either depression or anxiety in adjusted models. Considering reasons for the lack of associations between HTLV-1 and either depression or anxiety in their sample compared to findings from Brazil showing associations between HTLV-1 and depression and anxiety, the authors speculated that socioeconomic and cultural factors could account for their not finding associations between HTLV-1 and either depression or anxiety [22].

Indeed, much of the research investigating associations between HTLV-1 and cognitive function and between HTLV-1 and other neuropsychiatric outcomes has used samples from low-to-middle-income regions where HTLV-1 is endemic, with little research investigating these associations in non-endemic, high-income regions. However, evidence suggests that differences in early-life factors such as exposure to stress, pollution, and infectious diseases might affect outcomes to later infectious diseases [23], suggesting that a more complete characterization of associations between HTLV-1 and both cognitive and neuropsychiatric outcomes require the use of samples from both low-to-middle-income and high-income regions. In this study, therefore, we sought to generalize further previously reported associations between HTLV-1 and cognitive function and between HTLV-1 and depression by examining cognitive function and depression associated with HTLV-1 seropositivity and serointensity regardless of other symptomatology associated with HTLV-1 similar to the above study of Guiltinan et al. [22] This is a community-based sample of middle-aged to older middle-aged adults in a high-income region that likely differs on a number of sociocultural variables from regions where previous studies investigating associations between HTLV-1 and cognitive and neuropsychiatric outcomes have taken place. To do so, we used the UK Biobank sample while adjusting for an array of covariates that themselves could be associated with cognitive function and depression.

## 2. Results

Table 1 presents sample demographic and clinical characteristics of the HTLV-1 seropositive and seronegative groups. The data presented are for the largest analytic sample available for each variable. In the overall sample, fifty-five percent of the overall sample were women. The average age was 55.3 years, 95 percent were white, and 41 percent had obtained a college degree. One and a half percent were seropositive for HTLV-1 according to UK Biobank seropositivity criteria. The total sample size was 6785 with a sample size of 107 for the HTLV-1 seropositive group and 6678 for the HTLV-1 seronegative group. The average age of the HTLV-1 seropositive group was 57.598 years, and it was 55.265 years for the HTLV-1 seronegative group. Women comprised 40.2 percent of the HTLV-1 seropositive group and 54.8 percent of the HTLV-1 seronegative group. The percentage with a college degree was similar between the HTLV-1 seropositive and seronegative groups, as were income and self-rated health. The sample size of the HTLV-1 seropositive group with data for depression was only 33, and there was reasoning data for only 42 HTLV-1 seropositive participants.

In adjusted models, there were no associations between HTLV-1 seropositivity, the natural-log transformed concentration of the gag antibody, the natural-log transformed concentration of the env antibody, and the mean of the natural-log transformed concentrations of the env and gag antibodies and performance on reasoning, pairs matching, or reaction-time cognitive tasks or with depression (Table 2).

Of the 64 interaction models (Table 3, Table 4, Table 5 and Table 6), only three showed significant interactions: age interacted with the natural-log transformed concentration of the gag antibody concentration for performance on the pairs-matching task (Table 3), educational attainment interacted with HTLV-1 seropositivity for performance on the pairs-matching task (Table 5), and educational attainment interacted with the natural-log transformed concentration of env antibody for depression (Table 5). However, in none of these cases was the accompanying multivariate test significant, suggesting that these interactions are likely false positives.

## 3. Discussion

Previous findings primarily from regions endemic for HTLV-1 suggest that HTLV-1 is associated with cognitive impairment and depression, consistent with associations between HTLV-1 and several types of neurological disease, including HAM/TSP [10,17,19]. Despite previous findings associating HTLV-1 with worse cognitive function and depression, however, our main findings in this community sample of adults in the UK are that there were no associations of HTLV-1 seropositivity and serointensity with performance on three cognitive tasks—reasoning, pairs-matching, and reaction time or depression. We also sought to investigate whether certain groups might be vulnerable to cognitive deficits or to depression from HTLV-1 by examining interactions between age, sex, educational attainment, and income and HTLV-1. While we found three significant interactions—sex and educational attainment moderated the association between HTLV-1 and performance on the pairs-matching task, and educational attainment moderated the association between HTLV-1 and depression—we think that these statistically significant interactions were most likely due to chance from repeated testing and not meaningful in that only three out of 64 (4.7 percent) interaction models were statistically significant. Furthermore, multivariate tests designed to address alpha inflation also suggested that these were likely chance results. While the seroprevalence we found of 1.5 percent for HTLV-1 is higher than the estimates of 0.3 percent to 0.5 percent for HTLV in the UK population [12], the UK Biobank sample is not representative of the UK population and so cannot be used to determine prevalence [24]. However, our aim was not to determine the seroprevalence of HTLV-1 in the UK population; rather, we sought to investigate whether there was an association between evidence of exposure to HTLV-1 and cognitive function and depression, aims for which use of the UK Biobank data appear to be valid [24]. Because the UK Biobank sample is not designed to be representative of the UK population, it is difficult to make meaningful comparisons between the UK Biobank sample we used and the general UK population. The comparatively high seroprevalence of HTLV-1 in the sample we used compared to the general UK population and the lower percentage of women in the HTLV-1 seropositive group compared to the percentage of women on the HTLV-1 seronegative group [12] suggest that the sample we used is not representative of the general UK population). However, within our analytic sample, the HTLV-1-seropositive and HTLV-1-seronegative groups according to visual inspection appeared similar in mean age, percent White, percent who graduated from college, income, and mean self-rated health. The percentage of women was higher in the HTLV-1 seronegative group (54.8 percent) compared to the HTLV-1 seronegative group (40.2 percent).

Our findings showing no associations between HTLV-1 and cognitive function differ from several other previous reports that have shown associations between HTLV-1 and cognitive function and depression in comparisons of HAM/TSP with healthy controls and with asymptomatic HTLV-1 and healthy controls [10,11,19,21]. Several factors could plausibly account for the differences between our findings and previous reports that found associations between HTLV-1 and cognitive function and between HTLV-1 and depression. We were only able to examine potential associations between reasoning, pairs matching, and reaction time. Had we been able to examine associations between HTLV-1 and other tasks measuring cognitive function, particularly in other cognitive domains, it is possible that we would have identified statistically significant associations. In this regard, Gascón et al. [19] found that HTLV-1 was associated with worse cognitive function on some but not all measures of cognitive function. Further, although other studies have found that while HTLV-1 was associated with worse cognitive function, some but not all of the effect sizes were small [17]. The finding of no association between HTLV-1 and depression is consistent with the results of a study done in the US that also found no association with depression [22].

The large sample sizes we used that ranged from 2269 to 6785 in the analyses of cognitive function and 1673 in the analyses of depression suggest that our analyses were adequately powered to detect any associations between HTLV-1 and reasoning, pairs matching, and reaction time and between HTLV-1 and depression, arguing that in this sample there were indeed no associations between HTLV-1 and performance on these particular assessments of cognitive function and with depression. However, the small size (33) of the HTLV-1 seropositive group with depression and the small size of the HTLV-1 seropositive group who had data for reasoning could have lowered the power of our analyses to detect associations between HTLV-1 seropositivity and either depression or reasoning.

As ours was a community sample, it is likely that most of the people in our study did not have HAM/TSP, whereas some previously reported findings were from studies that had solely investigated HAM/TSP compared to healthy controls. In this regard, spinal-cord injury itself is associated with decreased cognitive function [25], and the proviral load is higher in HAM/TSP than in asymptomatic HTLV-1 [15]. However, previous findings also show that otherwise asymptomatic HTLV-1 is associated with worse cognitive function [19].

Another factor possibly accounting for potential geographical differences in the effects of HTLV-1 on cognition and depression is that there are different subtypes of HTLV-1 [12], which could possibly account for the lack of cognitive and depression differences between HTLV-1 seropositive and seronegative participants we found, although associations between subtypes of HTLV-1 and cognitive function and depression are not adequately explored.

Furthermore, while our study was based on people living in the UK, the majority of people with HTLV-1 in the UK, however, appear to be in people who have come from a region endemic for HTLV-1, who have had sexual contact with a person from an endemic region, or who are children of or descended from someone from an endemic region [12], suggesting that different HTLV-1 subtypes might not necessarily account for our not finding differences in cognitive function and in depression.

Differences in host factors other than the covariates we used in our models could also account for some of the differences between the results we found and other reports showing associations between HTLV-1 and worse cognitive function and depression, particularly because host immune factors are likely involved with differential responses to HTLV-1 [13]. Nonetheless, geographical region could be an important variable in determining whether HTLV-1 is associated with worse cognition or with depression.

Similarly, and of relevance to our findings showing no association between HTLV-1 and cognitive function and depression in the UK sample, evidence suggests that early-life events such as exposure to pollution, nutritional deficits, infection, and stress might be associated with persistent changes in immune function that could influence the outcomes of infectious diseases in adulthood. Adversity in early life might result in relative immune senescence that contributes to a proinflammatory state later in life that affects the immune response to subsequent infection [23]. As such, early-life adversity could affect associations between HTLV-1 infection and cognitive deficits and depression. While not providing direct evidence of this hypothesis, our findings of no association between HTLV-1 and cognitive function and between HTLV-1 and depression in a high-income nation that differ from previous findings from low-to-middle-income nations suggest that sociocultural and socioeconomic factors including early-life adversity might affect associations between HTLV-1 exposure in this case and later cognitive and neuropsychiatric outcome. An important caveat to this conclusion based on our findings is that the seroprevalence of HTLV-1 in the UK Biobank sample we used is very low, and previous findings suggest that many of the cases of HTLV-1 in the United Kingdom are found in immigrants from other regions, people who would have likely experienced different early-life sociocultural and socioeconomic factors from those experiences by much of the population in the United Kingdom. Even if this is correct, though, the sociocultural and socioeconomic factors experienced by these people after arrival in the United Kingdom may have resulted in different immune responses to HTLV-1, with resulting different effects on cognitive and depression outcomes. Furthermore, many of the participants in the UK Biobank who were seropositive for HTLV-1 could have acquired HTLV-1 in the United Kingdom. Finally, in the sample we used, educational attainment and income were similar between the HTLV-1 seropositive and HTLV-1 seronegative groups, indicating that the HTLV-1 seropositive group in our analyses was similar in many ways to the HTLV-1 seronegative group.

In addition to exposure to early-life events, differences in previous and comorbid infections in the sample we used compared to other previously reported samples also could be factors accounting for our not finding associations between HTLV-1 and either cognitive function or depression. Related to this issue are findings showing that coinfection with human immunodeficiency virus or hepatitis C virus is an independent risk factor for mortality in patients with HTLV-1 [26].

Somewhat consistent with our findings of no associations between HTLV-1 and cognitive function are findings from one small study showing no associations between HTLV-1 and HAM/TSP with brain volume [27], although another study found associations between HTLV-1 and ventricular volume and periventricular lucency [20].

Findings so far from investigating associations between HTLV-1 and cognitive function lead to several unanswered questions, including whether geographical region affects the association between HTLV-1 and worse cognition and depression. In addition, if HTLV-1 is indeed associated with worse cognitive function, an important question is whether it is associated with subsequent dementia, as cognitive deficits have been associated with later dementia [28], and because HTLV-1 itself was associated with vascular dementia in one study [20].

Despite not finding an association between HTLV-1 and cognitive function, our study has several strengths. The exposure variables are based on objective measures, as were the cognitive outcome variables. In addition, we included multiple covariates in the statistical models to account for possible confounding. The sample sizes were large, reducing the chances of finding false positives and false negatives. However, the study also has several limitations in addition to those we have already considered. For independent variables, we used measures of seropositivity and serointensity but not viral load, which could be better associated with cognitive function and depression than seropositivity and serointensity. Because of the cross-sectional data we used, we were unable to determine when the initial exposure to HTLV-1 might have occurred. It is feasible that different subgroups classified according to the time of initial exposure to HTLV-1 and to when during neural development the exposure occurred could be more or less susceptible to worse cognitive effects or to depression from HTLV-1 exposure.

In conclusion, we found no associations between HTLV-1 seropositivity and serointensity with cognitive function assessed by reasoning, pairs-matching, and reaction-time tasks and between HTLV-1 seropositivity and serointensity with lifetime depression in this community-based study of adults from the UK Biobank. The potential for geographical region and other variables to influence previously reported associations between HTLV-1 and cognitive function and between HTLV-1 and depression and the large number of people seropositive for HTLV-1 worldwide requires additional research into associations between HTLV-1 and cognitive function and between HTLV-1 and depression.

## 4. Materials and Methods

### 4.1. Study Sample

For this study, we used a subset of participants of the UK Biobank Resource, a large community-based sample of adults that received ethical approval from the National Research Ethics Service Committee North West-Haydock (reference 11/NW/0382) and consent from all participants (http://biobank.ctsu.ox.ac.uk/crystal/field.cgi?id=200, accessed on 10 September 2021). Upon application, the UK Biobank gave us approval to use deidentified data under application number 41535. The UK Biobank enrolled participants sampled from population-based registries (http://www.ukbiobank.ac.uk, accessed on 10 September 2021) and accessed at 22 centers in the United Kingdom [29]. Enrolling approximately 500,000 adults mostly between ages 40 and 70 years, the UK Biobank obtained clinical and demographic data via a variety of means, including biological samples, nurse interviews, physical examinations, and questionnaires (http://biobank.ctsu.ox.ac.uk/crystal/field.cgi?id=200, accessed on 21 October 2021). Although the UK Biobank sample was not designed to be representative of the UK population and even though there was a very low response rate to the initial surveys in the study, data from the UK biobank appear valid for establishing exposure-outcome associations [24].

We used different analytic samples for each outcome variable. Samples were limited to participants assayed for HTLV-1 and who had undergone cognitive testing or evaluation for depression and who had the preidentified covariates available (https://biobank.ctsu.ox.ac.uk/crystal/crystal/docs/infdisease.pdf, accessed on 21 October 2021). Across the study’s timeline, the UK Biobank includes a variety of neuropsychological tasks given to selected participants to assess cognitive function. However, the combination of the relatively small subgroup of the larger UK Biobank sample that were assayed for HTLV-1 and its low prevalence in the sample limited our cognitive analyses to the reasoning, pairs-matching, and reaction-time cognitive measures (i.e., the sample with both the HTLV-1 assay and cognitive measures data resulted in insufficient variation in HTLV-1 seropositivity for analyses of other cognitive assessments). Overall, there were 9430 participants who were assayed for HTLV-1. Most of these had valid data for the pairs-matching and reaction-time cognitive variables, but there were only about 3000 who had valid data for reasoning and approximately 2000 for depression. After removing observations with missing information on the covariates, the respective analyses included 2269 participants for reasoning, 6785 for pairs matching, 6757 for reaction time, and 1673 for depression.

### 4.2. Measures

#### 4.2.1. HTLV-1

The UK Biobank defined HTLV-1 seropositivity based on levels of HTLV-1 gag and env antibodies (https://biobank.ctsu.ox.ac.uk/crystal/field.cgi?id=23062, accessed on 21 October 2021) [30]. Respondents were considered seropositive for HTLV-1 if either their gag antibody titers were greater than 1500 or their env antibody titers were greater than 150. The sensitivity for gag alone is 96% and 88% for env alone, with a sensitivity of 94% for both gag and env. The specificity for gag alone is 97% and 100% for env alone. For both gag and env, the specificity is 100% [30]. This pattern, therefore, suggests that either gag or env antibody titers in the monoplex serology the UK Biobank used (https://biobank.ctsu.ox.ac.uk/crystal/field.cgi?id=23062, accessed on 21 October 2021) [30] are acceptable in determining HTLV-1 seropositivity. In addition to the HTLV-1 seropositivity variable, we estimated models that used HTLV-1 serointensity based on natural-log transformed concentrations of gag and env antibodies and on the mean of the natural-log transformed concentrations gag and env antibody standardized values.

#### 4.2.2. Cognitive Function

The reasoning task the UK Biobank used assesses fluid intelligence (higher score is better). In the pairs matching task that the UK Biobank used, participants attempt to remember the position of matching pairs of cards. Both the number of correct and incorrect matches were recorded. Because all participants were allowed to complete the task, almost all reached the total number of pairs (i.e., 6). Therefore, we only used the number of incorrect responses (lower score is better). Reaction time is the mean time of attempts to identify matching pairs of images (lower time is better). We included the natural log of reaction time in the analyses because reaction time was highly skewed. Further information about these cognitive tasks is available at https://biobank.ctsu.ox.ac.uk/crystal/label.cgi?id=100026, accessed on 10 September 2021).

#### 4.2.3. Depression

In assessing depression in the UK Biobank, we used methods outlined by Smith et al. [31] that included questions from the Patient Health Questionnaire [32], participant history of treatment for mood symptoms, and items from a screening measure administered by the UK Biobank (https://biobank.ctsu.ox.ac.uk/showcase/showcase/docs/TouchscreenQuestionsMainFinal.pdf, accessed on 10 September 2021) to identify participants with a lifetime history of major depression.

### 4.3. Covariates

We were particularly interested in including variables in our models that could potentially confound the associations of HTLV-1 with cognitive function or depression. To do so, we included covariates already associated with cognitive function [33] or that plausibly could be associated with cognitive function or depression. Accordingly, we included as covariates age (years), sex (female, male), race-ethnicity (white, nonwhite), educational attainment (college degree, less than college degree), income (the midpoint of categories for the following categories in 10,000 £/year: less than 18,000; 18,000 to 30,999; 31,000 to 51,999; 52,000 to 99,999; and 100,000 £ and above), self-rated health (four-point scale ranging from poor to excellent), body-mass index (kg/m^2^), smoking history (non-smoker, past, current), and alcohol use (six categories ranging from never to daily or almost daily).

### 4.4. Statistical Analysis

We explored the relationship of HTLV-1 seropositivity and serointensity with cognitive functioning and depression in a series of analyses. We used linear regression to model the relationship between HTLV-1 and cognitive functioning because the cognitive functioning dependent variables were continuous; we used logistic regression to model the association between HTLV-1 and depression as the depression variable was dichotomous. Table 1 presents four sets of adjusted models, one set for each measure of HTLV-1 (i.e., HTLV-1 seropositivity, natural-log transformed gag antibody concentration, natural-log transformed env antibody concentration, and the mean of the natural-log transformed gag and env antibody concentrations) as a focal predictor of each of the four outcomes (the three cognitive functioning measures and depression). Table 3, Table 4, Table 5 and Table 6 include a series of adjusted models that also include interactions between age, sex, educational attainment, and income, respectively, and measures of HTLV-1 for the four dependent variables to investigate whether certain groups might be more vulnerable to HTLV-1.

The thorough examination of the relationship of HTLV-1 with cognitive functioning and depression presented here requires a relatively large number of statistical tests, which also presents a heightened risk of identifying false positive results. To protect against this risk, we estimated multivariate tests that examined the relationship between a single focal predictor and the four dependent variables [34]. The tests were performed using Stata’s *suest* command [35], which produces a single parameter vector for the models included in the test that captures the joint covariance of the dependent variables. For example, the Multivariate p presented in Table 2 for the Seropositive row is a test of whether HTLV-1 seropositivity is jointly related to the four outcomes. The Multivariate p presented in Table 3 in the Seropositive Interaction row is a test of whether the interaction between HTLV-1 seropositivity and age is jointly related to the four outcomes. The null hypothesis for these tests is that the joint relationship between the predictor and the dependent variables is zero. Multivariate tests with *p*-values smaller than 0.05 were considered statistically significant.

We used Stata 17.0 (StataCorp, Stata Statistical Software, Release 17.0. College Station, TX, USA) for all statistical analyses.

## Figures and Tables

**Table 1:** Descriptive statistics of study variables.

	HTLV-1 Seropositive					HTLV-1 Seronegative				
	Mean	SD	Min	Max	*N*	Mean	SD	Min	Max	*N*
Cognition										
Reasoning	6.238	1.897	3	11	42	6.367	2.094	0	13	2227
Pairs matching	4.084	4.439	0	29	107	4.095	3.325	0	39	6678
ln(Reaction time)	6.322	0.182	6	7	106	6.288	0.183	6	7	6651
Depression	0.212	0.415	0	1	33	0.281	0.450	0	1	1640
HTLV-1										
ln(gag antigen)	6.118	1.582	0	8	107	4.393	1.996	0	7	6678
ln(env antigen)	4.480	1.294	0	7	107	3.071	1.035	0	5	6678
Mean of ln(gag) and ln(env)	1.063	0.498	−1	3	107	−0.037	0.779	−3	1	6678
Age	57.598	7.838	40	69	107	55.265	8.133	40	70	6678
Female	0.402	0.493	0	1	107	0.548	0.498	0	1	6678
White	0.972	0.166	0	1	107	0.951	0.216	0	1	6678
College degree	0.393	0.491	0	1	107	0.407	0.491	0	1	6678
Income (in 10,000 lb)	4.500	3.246	1	12	107	4.498	3.083	1	12	6678
Self-rated health	2.888	0.705	1	4	107	2.931	0.727	1	4	6678
Body-mass index	26.485	4.012	20	42	107	27.173	4.738	16	61	6678
Smoking status										
Non-smoker	0.542	0.501	0	1	107	0.578	0.494	0	1	6678
Past	0.327	0.471	0	1	107	0.333	0.471	0	1	6678
Current	0.131	0.339	0	1	107	0.089	0.285	0	1	6678
Drinking frequency										
Daily or almost daily	0.299	0.460	0	1	107	0.226	0.418	0	1	6678
3–4 times/week	0.187	0.392	0	1	107	0.243	0.429	0	1	6678
Once or twice/week	0.280	0.451	0	1	107	0.246	0.431	0	1	6678
1–3 times/month	0.056	0.231	0	1	107	0.111	0.314	0	1	6678
Special occasions	0.103	0.305	0	1	107	0.111	0.314	0	1	6678
Never	0.075	0.264	0	1	107	0.063	0.243	0	1	6678

Abbreviations: SD = Standard deviation, Min = Minimum, Max = Maximum. Source: UK Biobank.

**Table 2:** Adjusted Models of Cognitive Functioning and Depression on HTLV-1.

	Reasoning ^a^	Pairs Matching ^a^	Reaction Time ^a^	Depression ^b^	Multivariate *p* ^c^
Seropositive	−0.081	−0.179	0.022	0.637	0.439
	[−0.664,0.503]	[−0.810,0.453]	[−0.011,0.055]	[0.264,1.533]	
ln(gag)	−0.017	0.012	−0.000	1.032	0.649
	[−0.057,0.022]	[−0.027,0.052]	[−0.002,0.002]	[0.976,1.091]	
ln(env)	−0.032	−0.009	−0.001	0.975	0.820
	[−0.104,0.041]	[−0.084,0.065]	[−0.005,0.002]	[0.881,1.080]	
Mean of ln(gag) and ln(env)	−0.055	0.012	−0.002	1.027	0.747
	[−0.153,0.044]	[−0.088,0.113]	[−0.007,0.003]	[0.895,1.180]	

Note: Each cell in the table represents the results from a separate model. The main independent variable is indicated by the row label and the dependent variable is listed in the column header. Each model is adjusted for age, sex, race-ethnicity, educational attainment, household income, self-rated health, body-mass index, smoking status, and frequency of drinking alcohol. ^a^ Point estimates are unstandardized coefficients from linear regression. ^b^ Point estimates are odds ratios from logistic regression. ^c^ The multivariate test is a test of the null hypothesis considered within the joint covariance of the dependent variables (i.e., Reasoning, Pairs matching, Reaction time, and Depression) that the measure of HTLV-1 (i.e., Seropositive, ln(gag), ln(env) and the Mean of ln(gag) and ln(env)) is related to the set of dependent variables. It is applied here to address potential problems of reporting false positives because of the number of statistical tests performed. Significant relationships between an HTLV-1 measure and the dependent variables are thus ignored if the probability of the multivariate null being true is greater than 0.05. Reasoning *N* = 2269. Pairs matching *N* = 6785. Reaction time *N* = 6757. Depression *N* = 1673.

**Table 3:** Adjusted Models of Cognitive Functioning on the Interaction Between HTLV-1 and Age.

	Reasoning ^a^	Pairs Matching ^a^	Reaction Time ^a^	Depression ^b^	Multivariate *p* ^c^
Seropositive					
HTLV-1	2.083	−3.015	0.044	0.766	
Age	0.002	0.065 ***	0.007 ***	0.969 ***	
Interaction	−0.039	0.049	−0.000	0.997	0.690
ln(gag)					
HTLV-1	0.101	−0.289 *	0.012	0.959	
Age	0.011	0.041 ***	0.008 ***	0.962 *	
Interaction	−0.002	0.005 *	−0.000	1.001	0.101
ln(env)					
HTLV-1	0.268	−0.160	0.002	0.824	
Age	0.018	0.057 ***	0.007 ***	0.960 *	
Interaction	−0.005	0.003	−0.000	1.003	0.798
Mean of ln(gag) and ln(env)					
HTLV-1	0.406	−0.612	0.021	0.793	
Age	0.001	0.066 ***	0.007 ***	0.969 ***	
Interaction	−0.008	0.011	−0.000	1.005	0.231

Note: Each main effects and interaction set represents the results from a separate model. The dependent variable is listed in the column header. Each model is adjusted for sex, race-ethnicity, educational attainment, household income, self-rated health, body-mass index, smoking status, and frequency of drinking alcohol. ^a^ Point estimates are unstandardized coefficients from linear regression. ^b^ Point estimates are odds ratios from logistic regression. ^c^ The multivariate test is a test of the null hypothesis considered within the joint covariance of the dependent variables (i.e., Reasoning, Pairs matching, Reaction time, and Depression) that the interaction between age and the measure of HTLV-1 (i.e., Seropositive, ln(gag), ln(env), or the Mean of ln(gag) and ln(env)) is related to the set of dependent variables. It is applied here to address potential problems of reporting false positives because of the number of statistical tests performed. Significant relationships between an HTLV-1 measure and the dependent variables are thus ignored if the probability of the multivariate null being true is greater than 0.05. Reasoning *N* = 2269. Pairs matching *N* = 6785. Reaction time *N* = 6757. Depression *N* = 1673. * *p* < 0.05, ** *p* < 0.01, *** *p* < 0.001.

**Table 4:** Adjusted Models of Cognitive Functioning on the Interaction Between HTLV-1 and Sex.

	Reasoning ^a^	Pairs Matching ^a^	Reaction Time ^a^	Depression ^b^	Multivariate *p* ^c^
Seropositive					
HTLV-1	−0.401	0.016	0.004	0.754	
Female	−0.261 **	0.019	0.032 ***	2.005 ***	
Interaction	0.672	−0.482	0.044	0.741	0.794
ln(gag)					
HTLV-1	0.009	0.013	0.000	1.019	
Female	−0.030	0.016	0.037 ***	1.815 *	
Interaction	−0.049	−0.000	−0.001	1.022	0.979
ln(env)					
HTLV-1	−0.001	−0.028	−0.004	0.924	
Female	−0.070	−0.091	0.018	1.502	
Interaction	−0.058	0.033	0.005	1.097	0.582
Mean of ln(gag) and ln(env)					
HTLV-1	0.013	−0.003	−0.003	0.960	
Female	−0.252 **	0.015	.032 ***	1.999 ***	
Interaction	−0.127	0.027	0.002	1.119	0.950

Note: Each main effects and interaction set represents the results from a separate model. The dependent variable is listed in the column header. Each model is adjusted for age, race-ethnicity, educational attainment, household income, self-rated health, body-mass index, smoking status, and frequency of drinking alcohol. ^a^ Point estimates are unstandardized coefficients from linear regression. ^b^ Point estimates are odds ratios from logistic regression. ^c^ The multivariate test is a test of the null hypothesis considered within the joint covariance of the dependent variables (i.e., Reasoning, Pairs matching, Reaction time, and Depression) that the interaction between sex and the measure of HTLV-1 (i.e., Seropositive, ln(gag), ln(env), or the Mean of ln(gag) and ln(env)) is related to the set of dependent variables. It is applied here to address potential problems of reporting false positives because of the number of statistical tests performed. Significant relationships between an HTLV-1 measure and the dependent variables are thus ignored if the probability of the multivariate null being true is greater than 0.05. Reasoning *N* = 2269. Pairs matching *N* = 6785. Reaction time *N* = 6757. Depression *N* = 1673. * *p* < 0.05, ** *p* < 0.01, *** *p* < 0.001.

**Table 5:** Adjusted Models of Cognitive Functioning on the Interaction Between HTLV-1 and College Degree.

	Reasoning ^a^	Pairs Matching ^a^	Reaction Time ^a^	Depression ^b^	Multivariate *p* ^c^
Seropositive					
HTLV-1	0.071	−0.772	0.029	0.825	
College Degree	0.968 ***	−0.136	−0.006	1.075	
Interaction	−0.526	1.511 *	−0.019	0.327	0.105
ln(gag)					
HTLV-1	−0.027	0.018	−0.000	1.037	
College Degree	0.859 ***	−0.047	−0.004	1.127	
Interaction	0.023	−0.015	−0.001	0.987	0.630
ln(env)					
HTLV-1	0.002	−0.045	−0.003	1.058	
College Degree	1.237 ***	−0.384	−0.020	2.077 *	
Interaction	−0.090	0.088	0.004	0.803 *	0.536
Mean of ln(gag) and ln(env)					
HTLV-1	−0.040	−0.008	−0.003	1.114	
College Degree	0.959 ***	−0.110	−0.007	1.058	
Interaction	−0.035	0.051	0.003	0.822	0.641

Note: Each main effects and interaction set represents the results from a separate model. The dependent variable is listed in the column header. Each model is adjusted for age, sex, race-ethnicity, household income, self-rated health, body-mass index, smoking status, and frequency of drinking alcohol. ^a^ Point estimates are unstandardized coefficients from linear regression. ^b^ Point estimates are odds ratios from logistic regression. ^c^ The multivariate test is a test of the null hypothesis considered within the joint covariance of the dependent variables (i.e., Reasoning, Pairs matching, Reaction time, and Depression) that the interaction between educational attainment and the measure of HTLV-1 (i.e., Seropositive, ln(gag), ln(env), or the Mean of ln(gag) and ln(env)) is related to the set of dependent variables. It is applied here to address potential problems of reporting false positives because of the number of statistical tests performed. Significant relationships between an HTLV-1 measure and the dependent variables are thus ignored if the probability of the multivariate null being true is greater than 0.05. Reasoning *N* = 2269. Pairs matching *N* = 6785. Reaction time *N* = 6757. Depression *N* = 1673. * *p* < 0.05, ** *p* < 0.01, *** *p* < 0.001.

**Table 6:** Adjusted Models of Cognitive Functioning on the Interaction Between HTLV-1 and Income.

	Reasoning ^a^	Pairs matching ^a^	Reaction time ^a^	Depression ^b^	Multivariate *p* ^c^
Seropositive					
HTLV-1	−0.632	−0.641	0.024	0.510	
Income	0.088 ***	−0.009	−0.004 ***	0.924 ***	
Interaction	0.124	0.103	−0.000	1.055	0.297
ln(gag)					
HTLV-1	−0.029	0.027	0.000	1.096	
Income	0.080 *	0.007	−0.003	0.986	
Interaction	0.003	−0.003	−0.000	0.986	0.345
ln(env)					
HTLV-1	−0.008	−0.104	−0.002	1.024	
Income	0.108 **	−0.072	−0.004	0.957	
Interaction	−0.005	0.021	0.000	0.989	0.606
Mean of ln(gag) and ln(env)					
HTLV-1	−0.052	−0.044	−0.001	1.182	
Income	0.091 ***	−0.007	−0.004 ***	0.923 ***	
Interaction	−0.001	0.012	−0.000	0.967	0.640

Note: Household income is measured in $10,000. Each main effects and interaction set represents the results from a separate model. The dependent variable is listed in the column header. Each model is adjusted for age, sex, race-ethnicity, educational attainment, self-rated health, body-mass index, smoking status, and frequency of drinking alcohol. ^a^ Point estimates are unstandardized coefficients from linear regression. ^b^ Point estimates are odds ratios from logistic regression. ^c^ The multivariate test is a test of the null hypothesis considered within the joint covariance of the dependent variables (i.e., Reasoning, Pairs matching, Reaction time, and Depression) that the interaction between income and the measure of HTLV-1 (i.e., Seropositive, ln(gag), ln(env), or the Mean of ln(gag) and ln(env)) is related to the set of dependent variables. It is applied here to address potential problems of reporting false positives because of the number of statistical tests performed. Significant relationships between an HTLV-1 measure and the dependent variables are thus ignored if the probability of the multivariate null being true is greater than 0.05. Reasoning *N* = 2269. Pairs matching *N* = 6785. Reaction time *N* = 6757. Depression *N* = 1673. * *p* < 0.05, ** *p* < 0.01, *** *p* < 0.001.

## Data Availability

Anonymized data from the UK Biobank is available through application http://www.ukbiobank.ac.uk (accessed 12 March 2020).
